# Detroit Lancet

**Published:** 1878-02

**Authors:** 


THE "DETROIT MEDICAL AND SURGICAL JOURNAL" has been discontinued. There will be issued in its stead the "Detroit Lancet"—Editors, Drs. H. A. Cleland and Leartus Connor.

